# Geographic and area-level socioeconomic variation in cardiometabolic risk factor distribution: a systematic review of the literature

**DOI:** 10.1186/s12942-018-0165-5

**Published:** 2019-01-08

**Authors:** Renin Toms, Andrew Bonney, Darren J. Mayne, Xiaoqi Feng, Ramya Walsan

**Affiliations:** 1 School of Medicine, University of Wollongong, Wollongong, Australia; 2 Illawarra Health and Medical Research Institute, Wollongong, Australia; 3 Public Health Unit, Illawarra Shoalhaven Local Health District, Warrawong, Australia; 4 Population Wellbeing and Environment Research Lab (PowerLab), School of Health and Society, Faculty of Social Sciences, University of Wollongong, Wollongong, Australia

**Keywords:** Cardiometabolic risk factors, Area-level socioeconomic disadvantage, Geographic variation

## Abstract

**Introduction:**

A growing number of publications report variation in the distribution of cardiometabolic risk factors (CMRFs) at different geographic scales. A review of these variations may help inform policy and health service organisation.

**Aim:**

To review studies reporting variation in the geographic distribution of CMRFs and its association with various proxy measures of area-level socioeconomic disadvantage (ASED) among the adult ( ≥ 18 years) population across the world.

**Methods:**

A systematic search for published articles was conducted in four databases (MEDLINE (Ovid), PubMed, Scopus and Web of Science) considering the interdisciplinary nature of the review question. Population-based cross-sectional and cohort studies on geographic variations of one or more biological proxies of CMRFs with/without an analysed contextual association with ASED were included. Two independent reviewers screened the studies and PRISMA guidelines were followed in the study selection and reporting.

**Result:**

A total of 265 studies were retrieved and screened, resulting in 24 eligible studies. The review revealed reports of variation in the distribution of CMRFs, at varying geographic scales, in multiple countries. In addition, consistent associations between ASED and higher prevalence of CMRFs were demonstrated. The reports were mainly from industrialised nations and small area geographic units were frequently used.

**Conclusion:**

Geographic variation in cardiometabolic risk exists across multiple spatial scales and is positively associated with ASED. This association is independent of individual-level factors and provides an imperative for area-based approaches to informing policy and health service organisation. The study protocol is registered in *International prospective register of systematic reviews* (Register No: CRD42018115294) PROSPERO 2018.

**Electronic supplementary material:**

The online version of this article (10.1186/s12942-018-0165-5) contains supplementary material, which is available to authorized users.

## Introduction

Cardiovascular disease (CVD) associated metabolic risk factors represent major global public health concerns. CVD is the leading cause of human death, accounting for 17.7 million (31%) of the 56.4 million total deaths reported worldwide in 2015 [1]. Coronary heart disease (7.4 million) and stroke (6.7 million) were responsible for the greatest mortality within CVD and have remained the leading cause for mortality for the last 15 years [2]. CVD and its associated metabolic risk factors are listed in the top 15 causes of disability adjusted life years (DALY) globally [3]. In keeping with historical trends, deaths due to CVD are projected to increase steeply and reach more than 23.6 million annually by 2030 [4].

An important way to control CVD is by focussing on reducing associated metabolic risk factors. In low resource settings, vulnerable and disadvantaged groups are more likely to be exposed to unhealthy products and practices and develop metabolic risk factors for the development of CVD [5]. Cardiometabolic risk factors (CMRFs) such as diabetes mellitus (DM), hyperlipidaemia, high body mass index (BMI), and chronic kidney disease (CKD) can predispose and worsen CVD. Individual level approaches to prevent and control these risk factors have demonstrated limited success as evidenced by its increasing rates [6–8]. Thus it is important, in addition, to discern the contextual associations of development of these risk factors to assist in mitigating this global epidemic.

Geographic inequalities in the distribution of CMRFs at varying scales are reported in multiple studies from different countries in association with area-level socioeconomic disadvantage (ASED). Reviewing the area level distribution patterns and associated area level disadvantages reported in these studies may deepen our understanding of the higher prevalence of CMRFs in some geographic areas. Most recent relevant reviews in this area have broadly covered the influence of physical, social and service environment characteristics on CVD risk [9–12]. However, the potentially important influence of ASED is critically under-examined. Systematic synthesis of evidence regarding this globally reported variation and association can inform policy development and healthcare service planning to detail area level approaches, in addition to individual level measures, to prevent and control CMRFs effectively.

Therefore, the questions attempted to answer in this review are: *Is there any geographic variation in the distribution of CMRFs among the adult population (aged 18* *years and above) across the world, and is this variation associated with ASED?* The studies expected to include were epidemiological or population based cross sectional and/or cohort studies.

## Methods

A review protocol was developed and registered in International prospective register of systematic reviews, PROSPERO 2018 (Register No: CRD42018115294) Available from: http://www.crd.york.ac.uk/PROSPERO/display_record.php?ID=CRD42018115294.

**Fig. 1:**
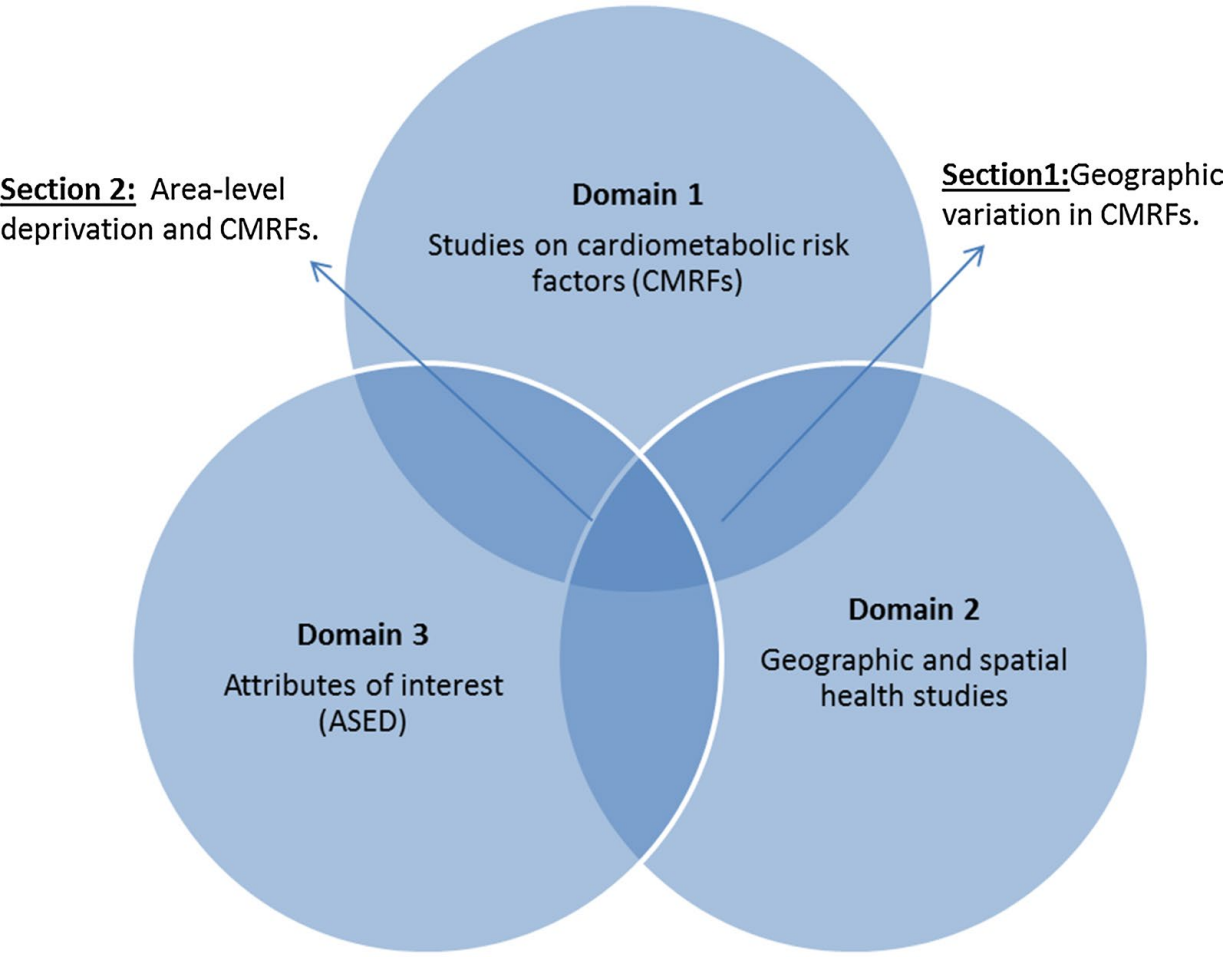
Conceptual representation of the literature search strategy

Four databases; MEDLINE (Ovid), PubMed, Scopus and Web of science databases were chosen for the search, considering the breadth of fields they cover and the interdisciplinary nature of the review question. Also, hand-searches of related articles served as ‘other sources’ of studies. The database search strategy commenced with two general search domains [1]: studies on CMRFs in singular and composite forms; and [2] geographic and spatial health studies. An intersectional retrieval of studies from both these domains yielded a narrower list of studies on geographic variation in CMRFs. A third domain [3] studies addressing area-level measures of socioeconomic disadvantages were further intersected with the retrieved studies to create a focal list of studies addressing geographic association of CMRFs with ASED. This approach maximised the number of potentially eligible studies identified compared to using single domain searches. Figure 1 conceptualizes the major search domains and their intersections used in the review.

The review included epidemiological or population-based cross-sectional and cohort studies on: geographic variation of one or more biological proxies of CMRFs, with/without an analysed contextual association with ASED. Obesity, diabetes mellitus (DM), hyperlipidaemia, and indices of low kidney function were the included biological proxies of CMRFs. Hypertension is included only when reported with other biological proxies of CMRFs, but not independently considering its limited summation into an overall cardiometabolic risk in an individual. Studies involving type 1 DM and gestational DM were excluded as they were out of scope for the current review pertaining to the geographic or area based contexts of the CMRFs. Studies measuring area-level characteristics other than ASED were also excluded.

All search outcomes were limited to: human studies; adult population (≥ 18 years); and availability in English language. The initial search included studies from year 1995; and latter it was modified to 01/01/2001 due to minimal publications on the review topic between the years 1995–2000. The search was last updated on 30/11/2018. Adopted search strategy in Ovid MEDLINE, and search result URLs of remaining databases are available in Additional file 1.

All retrieved studies were screened by two independent reviewers (RT and RW) in three stages to reduce the risk of bias. In stage 1, articles from all databases were combined and screened to remove duplicates. Titles and abstracts of remaining articles were screened for eligibility in stage 2. The final stage of study selection was done after full text reading of the remaining studies. Qualities of the individual studies were assessed using the STROBE checklist for cohort, case–control and cross-sectional studies (www.strobe-statement.org). The second coder repeated all three stages in parallel, and selected studies were matched at the conclusion of each stage and any differences were resolved by consensus and arbitration. Other review team members (AD, DJM and XF) served as additional reviewers when required.

Data extraction and coding of the chosen studies were carried out using two pilot-tested templates for consistency. Template 1 focused on the geographic variation in CMRFs and was used to extract information on author, year, nation, study design, sample size and characteristics, geographic unit of reporting, studied CMRFs, and the study outcome. Data on behavioural risk factors were not extracted as these were not included in the current review. Template 2 addressed the association of ASED and cardiometabolic risk prevalence, and extracted additional data on the reported proxies of ASED and its association status. An additional template was used for thematic mapping of the data in included studies for further qualitative syntheses. Study origin, representation, nature of problem, ecological context, and evidence strength were the mapped themes.

The two independent review authors extracted and coded the data, and any discrepancies were resolved through discussions between the authors. Summary measures used in this review are descriptive and based on the frequency of relevant studies to its denominator. Endnote software was used to keep track of the bibliographic details of the studies throughout the selection and data extraction process.

## Results

A total of 265 individual studies were retrieved from four electronic databases (n = 251) and hand searches of reference lists (n = 14). Studies from electronic data bases included 91 Ovid Medline, 80 PubMed, 58 Scopus, and 22 Web of science publications.

**Fig. 2:**
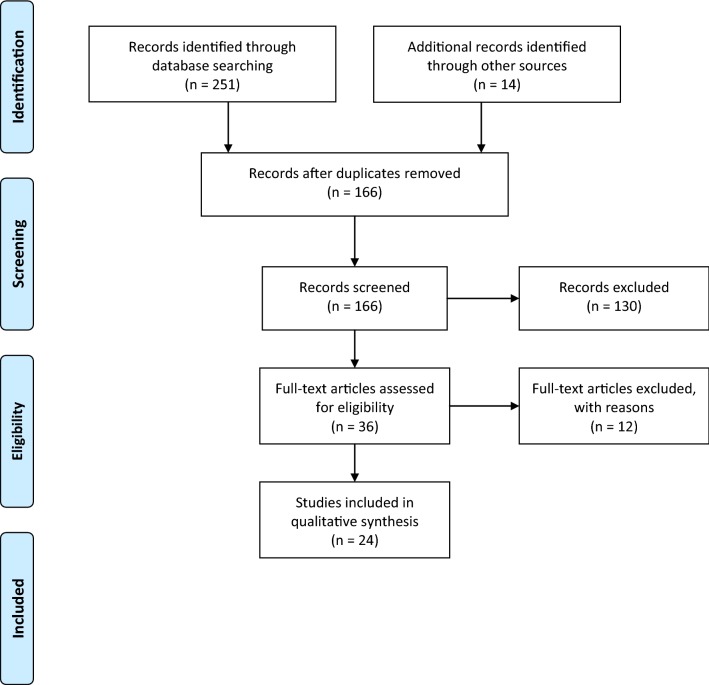
Combined PRISMA flow chart of the study selection

Figure 2 shows the screening process as per the Preferred Reporting Items for Systematic Reviews and Meta-Analyses PRISMA guidelines (www.prisma-statement.org) [13]. Stage 1 screening combined studies from all sources and removed the duplicates (n = 99). Duplicates in removed order: Ovid Medline (n = 0); PubMed (n = 80); Scopus (n = 10); Web of science (n = 3); and hand-searches (n = 6). After removing duplicates, 166 studies were forwarded for stage 2 screening. Stage 2 screening excluded 130 studies based on title and abstract screens, forwarding 36 studies for the full text screen. Studies excluded in stage 2 mainly addressed genetic, cellular, instrumental or pharmacological research regarding CMRFs. Studies on type 1 DM, paediatric or juvenile DM and gestational DM were also excluded at this stage as per the exclusions stated. Stage 3 screening carefully considered the whole full text of articles and 12 records were excluded with reason (list available in Additional file 2) leaving 24 studies for the systematic synthesis. PRISMA 2009  guidelines are followed in reporting the review and the checklist available in Additional file 3.

The review is structured into three sections. Screened research articles retrieved through ‘AND’ intersections of search domain 1 and 2 (n = 8) are reviewed in “Introduction” section: Geographic variation in the prevalence of cardiometabolic risk factors. Screened articles retrieved by intersecting domains 1 and 3 (n = 16) are reviewed in “Methods” section: Area level deprivation and cardiometabolic risk prevalence. Overall synthesis based on the total reviewed studies (n = 24) is presented in “Results” section: Overall synthesis of the studies.

### Geographic variation in the prevalence of cardiometabolic risk factors

Table 1 summarizes the eight studies reviewed under this section [13–20]. Geographic variation in the prevalence of one or more CMRFs is reported in each of these studies. Most of the studies (7/8) reported hyperglycaemia as an important biomarker displaying geographic variation in cardiometabolic risk [13–18, 20,] followed by dyslipidaemia (4/8), body mass index (4/8), blood pressure (BP) (3/8) and reduced glomerular filtration rate (GFR) (1/8).

**Table 1:** List of studies reviewed on geographic variation in CMRFs

	First author Country	Sample Age group	Design Geographic units	CMRFs^a^ (data source)	Outcome
1	Lawlor et al. UK [19]	4286 (women) 60–79 years	Cross-sectional 4 regions within country	HT, BMI, LDL, TC (data collected)	Geographic variation
2	Barker et al. USA [20]	813,498 DM ≥18 years	Cross-sectional 644 counties in proximity	DM prevalence ≥ 11.0%, (behavioral risk factor surveillance)	Geographic diabetic belt
3	Valdes et al. Spain [18]	5103 adults ≥ 18 years	Cross-sectional 2 region within country	BP, BMI, FPG,TC, WC (Di@bet.es study)	Geographic coherence
4	Astell-Burt et al. Australia [37]	114,755 adults ≥ 45 years	Cross-sectional ~ 40 local government areas (2011 significant urban boundary)	DM (the 45 and up study)	Geographic variation
5	Zhou et al. China [17]	98,058 adults > 18 years	Cross-sectional multilevel 31 provinces in country	DM (National Health Survey)	Geographic variation
6	Paquet et al. AU-France [16]	Au: 3893 (≥ 18 years) Fr: 6430 (30–79 years)	Cross-sectional multilevel Au: 767 CDs (SS, POA, SLA LGA). Fr: 1866 IRIS (TRIRIS, Municipalities)	BP,BMI,WC,FG,HbA1c,HR, TC,HDL, TG, (Au: NWAHS study, Fr: RECORD cohort study)	Geographic clustering
7	Alkerwi et al. Luxemberg [15]	1432 subjects 18–69 years.	Cross-sectional multilevel 106 municipalities (12 cantons)	BMI, FPG,TC, GFR (ORISCAV-LUX national survey)	Geographic variation
8	Oh et al. South Korea [13]	228,921 people ≥ 19 years	Cross-sectional 230 administrative districts	HT, DM (Korean Community Health Surveys)	Geographic clustering

*AU* Australia, *CD* census collection district, *POA* postal area, *SLA* statistical local area, *LGA* local government area, *IRIS* Ilôts regroupés pour l’information statistique, *TRIRIS* groups of around three IRIS areas, *BP* blood pressure, *BMI* body mass index, *DM* diabetes mellitus, *FBG* fasting plasma glucose, *FPG* fasting glucose, *HbA1c* glycated haemoglobin, *HR* heart rate, *HT* hypertension, *TC* total cholesterol; *TG* triglycerides, *LDL* low density lipoprotein, *GFR* glomerular filtration rate, *WC* waist circumference

^a^Behavioural risk factors excluded

All studies reported geographic variation in the prevalence of CMRFs, regardless of the geographic unit of analysis used [13–20]. Most of these studies were from Europe (4/8), predominantly from Western Europe (3/8) [15, 16, 18, 19]. These reports were from UK, [19] Spain, [18] France, [16] and Luxembourg [15]. In the UK, geographic variation in the prevalence of risk factors such as obesity, smoking, diabetes, hypertension and high cholesterol were reported across four main regions: South England: Midlands: and Wales: Scotland: and North England [19]. A higher prevalence of CMRFs was reported in southern Spain (Andalusia), which was found in close association with sedentary lifestyle and markers of socioeconomic disadvantage [18]. Variation in the distribution of diabetes, high BMI (≥ 25 kg/m^2^), abdominal obesity, hypertension, high cholesterol and low glomerular filtration rate were reported at both canton and municipality levels in Luxemburg, Western Europe [15].

BMI and resting heart rate were reported to have greater geographic variation among matched cohorts in France and Australia [16].

Other reports in this section were from Oceania (2/8), East Asia (2/8) and North America 1/8)—sourced from Australia, China, South Korea and US [13, 14, 16, 17, 20]. A geographic variation of 42% was reported in the odds of being diagnosed with DM among adults in Sydney, Australia [14]. In another Australian metropolitan based cohort, glycated haemoglobin (HbA1c) was reported to have geographic variation among matched cohorts in Australia and France [16]. In China, significant variation in the regional prevalence of diabetes was reported after adjusting for age, sex and urban/rural socioeconomic circumstances [17]. Geographic clustering of cardiometabolic risk factors were reported at administrative district level in South Korea [13]. The presence of a ‘diabetic belt’ with higher prevalence of diagnosed diabetes (> 11.0%) was reported in the United States, consisting of 644 counties in its 15 mostly southern states [20]. Though the risk profiles and parameters varied, all these studies consistently reported geographic variation in its CMRFs.

The geographic scales of area-based units reported in all these studies ranged from large regions [17–19] within countries to smaller jurisdictional administration units [13–16, 20] and trended towards smaller geographic areas over time. Easily accessible pre-existing geographic units and boundaries were used in these studies but most weren’t explicit on the spatial extention and average population within their geographic units. Three studies had relied only on self-reports on anthropometric, behavioural, biochemical, physiological and diagnostic categories of data, risking for recall bias and misclassifications [13, 14, 20].

### Area level deprivation and cardiometabolic risk prevalence

Table 2 summarises the 16 studies reviewed under this section [21–36]. Reported studies were mainly from Europe (7/16) and North America (7/16), followed by Oceania (1/16) and South America (1/16). Studies from Europe were predominantly reported from the western region and sourced from UK, Germany, Czech Republic and France. Reports from North America were mainly from USA (6/7) and Canada (1/7). There was only one study from Oceania, sourced from Australia [26]. Most of these studies were sourced from industrialised nations, except one study from Brazil, [21] a developing nation in South America.

**Table 2:** List of studies reviewed on the association of area-level deprivation and cardiometabolic risk prevalence

	First author Country	Sample Age group	Design Spatial unit	CMRFs* (data source)	Proxies of ASED (data source)	Association
1	Bonney et al. Australia [26]	91,776 adults 55.2 ± 15.66	Cross-sectional higherarchical 631 census collection districts	BMI (the SIMLR study)	Index of Relative Socioeconomic Disadvantage (Australian Census 2006)	+ve (women)
2	Unger et al. USA [27]	5805 adults 45–84 years	Prospective cohort higherarchical Census tract level	BMI, BP, BS, TC- CVH score (The MESA study)	Neighbourhood SES (constructed summary score)	+ve
3	Maier et al. Germany [29]	33,690 adults < 30 years	Cross-sectional design 412 districts	T2DM, obesity (GEDA national health interview survey)‘	German Index of Multiple Deprivation score (assessed by GIMD)	+ve (women)
4	Silhol et al. France [33]	19,808 adults 35–50 years	Cross-sectional cohort Municipality level	Incidence of CHD (French GAZEL cohort Data)	Area socio - economic position (French Census 1990)	−ve
5	Naimi et al. Canada [36]	342 adults 18–55 years	Cross-sectional 7 census tracts	BMI, HbA1c, TG, TC, HDL—TCR (Montreal Neighbourhood Survey of Lifestyle and Health)	Area-level unemployment (Canada Census 2001)	+ve
6	Cox et al. Scotland [24]	3917 adults < 35 years	Cross-sectional 3382 census output areas (OA)	T2DM (DARTS Diabetes Audit and Research Tayside Scotland dataset)	Area deprivation (The Carstairs score based on 2001 Scotland census data)	+ve
7	Andersen et al. UK [35]	4286 women 60–79 years	Cross-sectional 457 British electoral wards	T2DM, FBG, IR (British Women’s Heart and Health Study)	Area deprivation (The Carstairs score based on 2001 census data)	+ve
8	Gabert et al. USA [25]	63,053 DM 18–74 years	Retrospective observational 120 zip code areas	BP, HbA1c, LDL (Minnesota Community Measurement electronic health records)	Area-level indicators of SES (based on American Community Survey 2013)	+ve
9	Dragano et al. GR-Czech [32]	GR: 4814 adults CZ: 8856 adults 57.7 ± 6.6 years	2 longitudinal cohort studies 326 pre-existing administrative units	Obesity, HT (GR: ‘Heinz Nixdorf Recall (HNR) Study’, Czech: ‘Health, Alcohol and Psychosocial Factors in Eastern Europe (HAPIEE) Study’)	Area-level socioeconomic status (based on census data)	+ve
10	Cubbin et al. Sweden [31]	18,081 adults 25–64 years	Pooled cross-sectional data 8624 SAMS neighbourhoods	Obesity, DM, HT (Swedish Annual Level of Living Survey (SALLS), 1988–89)	Neighbourhood deprivation (assessed by Care Need Index (CNI) 1997 data)	+ve
11	Mujahid et al. USA [28]	13,167 adults 45–64 years	Crosssectional and longitudinal (3–9 years) Census block	BMI (The Atherosclerosis Risk in Communities ARIC Study)	Neighbourhood SES score (1990 U.S. Census1990)	−ve
12	Lawlor et al. UK [34]	4286 women 60–79 years	Cross-sectional 457 electoral wards	Coronary heart disease (British Women’s Heart and Health Study)	Residential area deprivation(The Carstairs score based on 1991 UK census data)	+ve
13	Roux et al. USA [30]	3093 adults 28–40 years	Cross-sectional 10 years follow up 2260 census block (in 45 states).	BMI, HDL, TG, BP, FI and FG -IRS (Coronary Artery Risk Development in Young Adults CARDIA Study)	Neighbourhood SES score (1990 U.S. Census)	−ve
14	Keita et al. USA [22]	19,079 black/white age > 45 years	Cross-sectional cohort Census block group	Obesity, WC, BP, FBG, TG, low-HDL (REGARDS study).	Neighborhood socioeconomic deprivation(US Census 2000)	+ve (black/white)
15	Clark, et al. USA [23]	3909 Afro-Americans 35–84 years	Cross-sectional cohort 102 census tracts	TG, FBG, BP, WC, low-HDL (Jackson Heart Study).	Neighborhood socioeconomic disadvantage (US Census 2000)	+ve (women)
16	Barber et al. Brazil [21]	10617 adults 35–75 years	Cross sectional cohort Study defined clusters of contiguous census tracts	DM and HT (Brazilian Longitudinal Study of Adult Health)	Area level economic residential segregation (IBGE census 2010)	+ve

*BMI* body mass index, *BP* blood pressure, *BS* blood sugar, *CHD* coronary heart disease, *CVD* cardiovascular disease, *CVH* cardiovascular health, *DM* diabetes mellitus, *eGFR* estimated Glomerular filtration rate, *FBG* fasting blood glucose, *FG* fasting glucose, *FI* fasting insulin, *GR* Germany, *HbA1c* glycated haemoglobin, *HDL* high density lipoprotein, *HT* hypertension, *IR* insulin resistance, *IRS* insulin resistance syndrome, *LDL* low density lipoprotein, *SES* socioeconomic status, *TC* total cholesterol, *TCR* total cardiometabolic risk, *T2DM* type 2 diabetes mellitus, *TG* triglycerides, *SAMS* small area market statistics

All studies reported associations of higher prevalence of CMRFs with greater ASED [21–36]. Various measures of the biological proxies of CMRFs reported includ biochemical, anthropometric, physiologic, behavioural and diagnostic categories of data. Census sourced data on ASED were used in most of these studies (12/16), whereas other survey sourced data were used in the remaining studies (4/16) to construct summary scores or indices on ASED. The categories of measures used to calculate ASED in these studies were area-level proportions of: median income; education; occupation; housing; transport; dependent population; social class; social capital; environment; security; family structure; disability; internet access; and insurance coverage. A minimum of one category of these measures were used in all the studies [21–36].

The samples characteristics and variables considered were notably heterogeneous across studies. The sampling frame of most (7/16) of these studies were population based lists, however service provider (4/16) and employee (3/16) lists were also used. Two studies had used a combination of both population lists and service provider given lists [27, 30]. Though subjects in all studies used adult age limits (≥ 18 years), divergent age groups were sampled across all of the studies. Also gender [34, 35] and race [22, 23] specific sampling were used in two studies each. Heterogeneity of these sample characteristics makes a comparison and further quantitative synthesis difficult.

The samples were mostly accessed from existing study cohorts, laboratory databases, national surveys and audit lists. The sample size of studies ranged from 342 adults to a maximum of 91,776 adults, mostly larger in size. Census administration units were the most commonly used neighbourhood proxy, followed by other administrative units and electoral wards. Pre-existing geographic boundaries were mostly adopted to define the spatial unit, but their spatial extents of the unit of analyses were not stated in most of the studies.

### Overall synthesis of the studies

Significant features of the included studies were identified to aid synthesis of the findings. These features were the origin of the study, its representativeness, nature of the CMRFs studied, the ecological context and the strength of evidence presented. These features were then formulated into five themes, mapping the related data for further analyses (Table 3).

**Table 3:** Thematic mapping of data categories from all included studies

Theme	Study origin	Representation	Ecological context	Nature of problem	Evidence Strength
Data map	Reference Nation (status) Region	Sample frame Sampling Response or retention %	Geographic unit and/or ASED	Cardiometabolic risk nature	Data source Analyses
1.	Roux et al. [30] USA (Industrialised) North America	Population and service providers' lists 79% retention	Small area ASED: income, education, occupation	Biochemical, anthropometric Physiological	Self-report, PE, Specimen tests Statistical
2.	Lawlor et al. [19] UK (Industrialised) Western Europe	Service provider’s list Random 60% response	Large area	Biochemical, anthropometric, physiological	Self-report, PE, Specimen tests, MR Statistical
3.	Mujahid et al. [28] USA (Industrialised) North America	Population list1 Random 81% retention	Small area ASED: income, education, occupation	Anthropometric	Self-report, PE Statistical
4.	Lawlor et al. [34] UK (Industrialised) Western Europe	Service provider’s list Random 60% response	Median area ASED: employment, housing, transport, social class	Biochemical, anthropometric, physiological	Self-report, PE, Specimen tests, MR Statistical
5.	Cubbin et al. [31] Sweden(Industrialised) Northern Europe	Population list2 Random4 ~ 80% response	Small area ASED: population structure, education, unemployment etc.	Anthropometric, physiological, diagnostic: DM	Self-report Statistical
6.	Cox et al. [24] Scotland (Industrialised) Western Europe	Service provider’s list ~Purposive	Small area ASED: employment, housing, transport, social class	Diagnostic—T2DM	Medical record Spatial and Statistical
7.	Dragano et al. [32] GR-Czech(Industrialised) Western—Central Europe	Population list Random 56 and 55% responses	Small area ASED: unemployment overcrowding	Anthropometric, physiological	Self-report, PE Statistical
8.	Andersen et al. [35] UK (Industrialised) Western Europe	Service provider’s lists Random 60% response	Small area ASED: employment, housing, transport, social class	Biochemical	Self-report, PE, MR Statistical
9.	Naimi et al. [36] Canada (Industrialised) North America	Population list Stratified cluster sampling 15% response	Medium area ASED: Education employment	Anthropometric, biochemical	Self-report, PE, Specimen tests Statistical
10.	Barker et al. [20] USA (Industrialised) North America	Population list Random 50.6% response	Medium area	Anthropometric, biochemical	Self-report Statistical
11.	Silhol et al. [33] France (Industrialised) Western Europe	Employees lists ~purposive	Medium area ASED: higher job, education	Anthropometric, biochemical physiological	Self-report, Employers data, Insurance data Spatial and Statistical
12.	Keita et al. [22] USA (Industrialised) North America	Population list	Small area ASED: income, housing education and occupation	Biochemical, anthropometric physiological	Self-report, PE, Specimen tests Statistical
13.	Clark et al. [23] USA (Industrialised) North America 6	Population list Random	Medium area ASED :10 components	Biochemical, anthropometric physiological	PE, Specimen tests Statistical
14.	Valdes et al. [18] Spain (Industrialised) Southern Europe	Population list Cluster –random 54.6% response	Large area	Anthropometric, hysiological, biochemical	Self-report, PE, Specimen tests Statistical
15.	Astell-Burt et al. [14] Australia (Industrialised) Oceania	Population (insurance) lists Random	Medium area	Biochemical, physiological	Self-report Spatial and Statistical
16.	Unger et al. [27] USA (Industrialised) North America	Population and service provider’s list (~ purposive)	Medium area ASED: income, housing, education, occupation.	Anthropometric biochemical physiological	Self-report, PE, Specimen tests Statistical
17.	Maier et al. [29] Germany(Industrialised) West - Central Europe	Population list Random 29.1% response	Large area. ASED: income, employment, education, revenue, social capital, environment, security	Anthropometric Diagnostic—T2DM	Self-report Statistical
18.	Zhou et al. [17] China (Developing) East Asia	Population (survey) list Random 90.5% response	Large area	Anthropometric biochemical	Self-report, Specimen tests Statistical
19.	Bonney et al. [26] Australia (Industrialised) Oceania	Service provider’s list (~ purposive)	Small area. ASED: income, education, employment, family structure, disability, housing, transport and internet connection	Anthropometric	Medical record Statistical
20.	Gabert et al. [25] USA (Industrialised) North America 8	Employees list 83.6% response	Small area ASED: income, education, insurance	Biochemical	Medical record Spatial and Statistical
21.	Paquet et al. [16] AU-France (Industrialised) Oceania - West Europe	Australia: Population list (Random 13/49.4% response 3) France: Employees list (Purposive/83.6% response)	Small area	Anthropometric, biochemical, hysiological	PE, Specimen tests Spatial and Statistical
22.	Alkerwi et al. [15] Luxemberg (Industrialised) Western Europe	Population (survey) list stratified random 32.2% response	Medium area	Physiological, biochemical	Self-report, PE, Specimen tests Spatial and Statistical
23.	Oh et al. [13] South Korea (Developing) East Asia	Population (ministry) lists ~purposive	Medium area	Biochemical, physiological, diagnostic	Self-report Spatial and Statistical
24.	Barber et al. [21] Brazil (Developing) South America	Employees lists ~purposive	Large area ASED: income	Biochemical, anthropometric, physiological	Self-report, PE, Specimen tests Spatial and Statistical

We had plotted all the included studies to identify their global region of origin and the economic nature of the source country. Most of the studies published were from Europe (11/24), closely followed by America (9/12), (two studies were cross national, hence counted under both the nations and corresponding regions). Fewer publications were found from Oceania (3/24), and Asia (2/24). However no identified studies were from Africa. Studies from developing nations were fewer (3/24) compared with studies from industrialised nations (21/24). This emphasises a gap in related publications from Asia–pacific and African regions, especially from nations of developing and underdeveloped economies. The global representativeness of this review is hence limited, and the review findings may be more generalizable to industrialised nations.

The target populations for included studies are shown in Table 3. The sample frame of most of the studies were population based lists (13/24 studies), however service providers’ lists (5/24) and employees lists (3/24) were also used. Both population and service providers’ lists were used in three studies (3/24). All the population based studies used a random sampling technique to ensure the population representativeness. However, the response rates varied (15–90.5%) in these studies. Two studies had a response rate < 50%, suggesting a risk of responder bias despite a probability sampling method being employed [29, 36].

Ecological contexts of the included studies were analysed by extracting area level characteristics (Table 3). Area level units used in these studies extended from small areas (10/24), to medium areas (9/24) and large areas (5/24). Small area units were mostly based on census, administrative or zip code area with an average ~ 1000 residing population. Medium area units had an average ~ 5000 population and the large area units were mostly regions, provinces and districts. ASED gradients were based on area level measures of ranged from 1 to 7 measures, however single measures of income or overcrowding as an indirect proxy of ASED raised concerns regarding their comprehensiveness in comparison to aggregate measures of ASED.

The nature of CMRFs and the strength of evidence in relation to associations with outcomes were mapped by extracting data on the categories of CMRFs measured, the source of data and the mode of analyses (Table 3). Biological proxy categories of CMRFs were mostly biochemical (18/24), followed by anthropometric (18/24), physiologic (15/24), and diagnostic (4/24) in nature. Self-reported data on these categories of CMRFs had the highest risk for misclassification due to reporting bias or errors. Studies which adopted a combined mode of both statistical and spatial analyses provided a better ecological context of CMRFs than with statistical analyses alone.

## Discussion

ASED was repeatedly demonstrated to be associated with higher cardiometabolic risk. Higher ASED was consistently reported to have an association with cardiovascular risk; whereas lower ASED was associated with reduced cardiovascular risk. Such associations were often demonstrated independently of individual level characteristics such as socioeconomic status, education and duration of exposure to area. Type 2 diabetes and high body mass index (BMI) were reported to be more prevalent in disadvantaged areas. Related studies report that the type of neighbourhood food outlets [37–39], poor physical activity resources [39], individual perception of area level features [40] residential density and service availability [41] were all explanatory variables associated with cardiometabolic risk prevalence among people living in disadvantaged neighbourhoods.

Related systematic reviews published in this area of research investigate associations for different geographically distributed factors with CVD. Chaix (2009) reviewed the associations between neighbourhood social environments and CHD, and proposed a theoretical model of a mediating mechanism focussing on the social interactional environment [10]. Consistent associations of obesity or hypertension with lower levels of area socioeconomic status, urbanization, street intersection, accessibility to supermarkets, social cohesion, service availability and residential density; and higher levels of noise pollution and density of convenience stores, were reviewed and reported by Leal [11]. Frequent inverse associations of the common indices of ASED with childhood obesity were reported in the UK [9]. Consistent associations between socio economic disadvantage and central adiposity was reported by Slopen [12]. All these reviews report important methodological inadequacies and the need for further research in this area, which support the findings of the current review.

Recent advances in geographic information systems (GIS) and analytical approaches were utilised in the studies reporting geographic variation in CMRFs. These studies have demonstrated advances in various analytical tools and the potential for plotting area level risk parameters. Geocoding and mapping of existing large population based datasets has become feasible with newer computational tools through linking location data; such as map co-ordinates, addresses or postcodes [42]. These tools have the capacity to visually display area based factors, in contrast with traditional table and graph methods, and this has the potential to enhance impact on subsequent area level health care policy development and resource allocation [43–45]. In addition, systematic quantitative analyses are possible with these spatial tools which create opportunities to investigate the role of environmental factors in explaining any geographic aggregations beyond random effects [46].

National estimates of CVD have limited utility in informing prevention and management of CVD within discrete communities. The disease patterns at smaller areas may significantly differ from national and regional prevalence reports, thus small area analysis is important in order to understand local patterns and requirements [47]. Small-area level analyses also have the potential to reveal area level contexts and dependencies of CMRFs and such analyses can highlight areas for targeted preventive interventions.

CVD and its associated CMRFs continue to evolve as a major global health threat. It is the highest cause of mortality and the highest absorber of health care expenditure in many developed nations [6, 48, 49]. Once diagnosed, the ongoing costs of care and productivity loss due to consequent disability and premature death creates a large economic burden not only to the individual and family, but to the nation—especially when half the people dying are found to be in their prime productive years [50]. Thus, CVD and its associated metabolic risk factors emerge as a threat not only to human health and life, but to the sustainable development and economies of nations. Hence, improving public health program effectiveness in reducing CVD must be a research priority.

### Limitations

Firstly, the cross sectional nature of the reviewed studies precluded causative interpretations. Second, the global representativeness of the review is limited mainly due to publication gaps from Asia–pacific and African regions of the World. Third, the scope of our review excluded examination of behavioural, dietary and activity related risk factors and also other area level characteristics to focus only on the biological proxies of CMRFs. Fourth, methodological heterogeneity within the retrieved studies prohibited a meta-analytical synthesis of the findings. The sample characteristics, geographical scales and the CMRFs’ risk profiles varied substantially across the studies impeding any further quantitative synthesis.

### Recommendations and future directions

Finding geographic variation in CMRFs (if any) and its association with ASED may assist in understanding the contexts of risk. Such studies have the potential to inform contextual planning of interventions for prevention and management of cardiometabolic risk. However, most of the studies in this review do not report the spatial extents of their units of analysis. This is important as associations are likely to be different at different levels of aggregation, and limits the ability to assess the likelihood of spatial scale effects in these studies [22, 23] known as the Modifiable Areal Unit Problem [51, 52]. When data are aggregated to larger geographic units, small-area anomalies may be diluted or smoothed over [22]. Using smaller rather than larger area scales can help to reduce the likelihood of missing important small area anomalies [53]. Similarly, supplementing individual level data along with area level data could minimise group effects due to area level aggregation of data [53]. Leveraging both individual- and area-level data provides a more complete picture to inform planning, policy and practice [46, 53]. Future research directions should include hierarchical multilevel analyses to yield comprehensive picture of the contextual aspects of risk factors, to help aid both individual and area-level better preventive initiatives.

## Conclusion

Cardiometabolic risk distribution varied significantly across different geographic scales reported in multiple studies. In addition, there is strong evidence that area-level disadvantage is significantly associated with CMRFs, irrespective of individual-level characteristics. This review highlights the need for area-based preventive approaches in addition to individual-level approaches to prevent and control CMRFs and their consequent CVD outcomes.

## Additional files

**Additional file 1.** The search strategy and results URLs.

**Additional file 2.** List of excluded full text studies with reason.

**Additional file 3.** PRISMA 2009 systematic review content checklist.

## Abbreviations

AU: Australia
ASED: area-level socioeconomic disadvantage
BP: blood pressure
BMI: body mass index
BS: blood sugar
CD: census collection district
CHD: coronary heart disease
CMRFs: cardiometabolic risk factors
CKD: chronic kidney disease
CVD: cardiovascular disease
CVH: cardiovascular health
DM: diabetes mellitus
eGFR: estimated Glomerular filtration rate
FBG: fasting blood glucose
FG: fasting glucose
FI: fasting insulin
FPG: fasting plasma glucose
GFR: glomerular filtration rate
GR: Germany
HbA1c: glycated haemoglobin
HDL: high density lipoprotein
HR: heart rate
HT: hypertension
IR: insulin resistance
IRS: insulin resistance syndrome
IRIS: ilôts regroupés pour l’information statistique
LDL: low density lipoprotein
LGA: local government area
POA: postal area
PRISMA: preferred reporting items for systematic reviews and meta-analyses
SES: socioeconomic status
SLA: statistical local area
TC: total cholesterol
TCR: total cardiometabolic risk
T2DM: type 2 diabetes mellitus
TG: triglycerides
TRIRIS: groups of around three IRIS areas
WC: waist circumference
