# Supplementary material for: Geographic and area-level socioeconomic variation in cardiometabolic risk factor distribution: a systematic review of the literature
Source: Int J Health Geogr. 2019 Jan 8;18:1. doi: 10.1186/s12942-018-0165-5 (PMC6323718; doi:10.1186/s12942-018-0165-5)
Supplement: Supplementary file 1 — Additional file 1. The search strategy and results URLs. [file 12942_2018_165_MOESM1_ESM.docx]

**Additional file 1a: SEARCH STRATEGY**

**Database: Ovid MEDLINE(R) <2001 to November 30, 2018>**

Search Strategy:

--------------------------------------------------------------------------------

1 cardiometabolic.ti,ab,mp. (6616)

2 cardio metabolic.ti,ab,mp. (1069)

3 metabolic syndrome.ti,ab,mp. (42423)

4 metabolic risk.ti,ab,mp. (3856)

5 Geographic.ti,ab,mp. (69264)

6 Geospatial.ti,ab,mp. (1148)

7 Spatial.ti,ab,mp. (211880)

8 regional variation.ti,ab,mp. (3211)

9 area socioeconomic.ti,ab,mp. (147)

10 neighbo?rhood socioeconomic.ti,ab,mp. (658)

11 area poverty.ti,ab,mp. (38)

12 neighbo?rhood deprivation.ti,ab,mp. (346)

13 1 or 2 or 3 or 4 (49526)

14 5 or 6 or 7 or 8 9 or 10 or 11 or 12 (274223)

15 13 and 14 (166)

16 limit 15 to (english language and humans and yr="2001 -Current" and "all adult (19 plus years)") (91)

**Additional file 1b: SEARCH URLs**

I. **Database: Scopus <2001 to November 30, 2018>**

<https://www-scopus-com.ezproxy.uow.edu.au/results/results.uri?sort=plf-f&src=s&sid=ea1c0f3d085ba8ac5196ec70f956b0b0&sot=a&sdt=a&cluster=scolang%2c%22English%22%2ct%2bscoexactkeywords%2c%22Human%22%2ct%2c%22Humans%22%2ct%2c%22Adolescent%22%2cf%2c%22Nonhuman%22%2cf%2c%22Child%22%2cf&sl=364&s=%28+TITLE-ABS-KEY+%28+geographic+OR+geospatial+OR+spatial+OR+regional+AND+variation+%29+OR+TITLE-ABS-KEY+%28+area+AND+socioeconomic+OR+area+AND+socioeconomic+OR+neighbo%3frhood+AND+socioeconomic+OR+area+AND+poverty+OR+neighbo%3frhood+AND+deprivation+%29+AND+TITLE-ABS-KEY+%28+cardiometabolic+OR+%22cardio+metabolic%22+OR+%22metabolic+syndrome%22+OR+%22metabolic+risk%22+%29+%29+AND+PUBYEAR+%3E+2000&origin=searchhistory&txGid=ecc5b4b292f0fe563250cb74eb62adc7>

Search history

( TITLE-ABS-KEY ( geographic OR geospatial OR spatial OR regional AND variation ) OR TITLE-ABS-KEY ( area AND socioeconomic OR area AND socioeconomic OR neighbo?rhood AND socioeconomic OR area AND poverty OR neighbo?rhood AND deprivation ) AND TITLE-ABS-KEY ( cardiometabolic OR "cardio metabolic" OR "metabolic syndrome" OR "metabolic risk" ) ) AND PUBYEAR > 2000 AND ( LIMIT-TO ( LANGUAGE , "English" ) ) AND ( LIMIT-TO ( EXACTKEYWORD , "Human" ) OR LIMIT-TO ( EXACTKEYWORD , "Humans" ) OR EXCLUDE ( EXACTKEYWORD , "Adolescent" ) OR EXCLUDE ( EXACTKEYWORD , "Nonhuman" ) OR EXCLUDE ( EXACTKEYWORD , "Child" ) )

II. **Database: PubMed <2001 to November 30, 2018>**

<https://www-ncbi-nlm-nih-gov.ezproxy.uow.edu.au/pubmed/?term=(((Geographic%5BTitle%2FAbstract%5D+OR+Geospatial%5BTitle%2FAbstract%5D+OR+Spatial%5BTitle%2FAbstract%5D+OR+%E2%80%9Cregional+variation%E2%80%9D%5BTitle%2FAbstract%5D))+OR+(%E2%80%9Carea+socioeconomic%22%5BTitle%2FAbstract%5D+OR+%E2%80%9Cneighbourhood+socioeconomic%E2%80%9D%5BTitle%2FAbstract%5D+OR+%E2%80%9Carea+poverty%E2%80%9D%5BTitle%2FAbstract%5D+OR+%E2%80%9Cneighbourhood+deprivation%E2%80%9D%5BTitle%2FAbstract%5D))+AND+(cardiometabolic%5BTitle%2FAbstract%5D+OR+%E2%80%9Ccardio+metabolic%E2%80%9D%5BTitle%2FAbstract%5D+OR+%E2%80%9Cmetabolic+syndrome%E2%80%9D%5BTitle%2FAbstract%5D+OR+%22metabolic+risk%22%5BTitle%2FAbstract%5D)>

Filters activated: Publication date from 2001/01/01 to 2018/11/30, Humans, English, Adult: 19+ years.

III. **Database: Web of science <2001 to November 30, 2018>**

<http://apps.webofknowledge.com.ezproxy.uow.edu.au/summary.do?product=UA&doc=1&qid=31&SID=C3eZJBIA1SNizT9v93Z&search_mode=CombineSearches&update_back2search_link_param=yes>
