# Supplementary material for: Geographic and area-level socioeconomic variation in cardiometabolic risk factor distribution: a systematic review of the literature
Source: Int J Health Geogr. 2019 Jan 8;18:1. doi: 10.1186/s12942-018-0165-5 (PMC6323718; doi:10.1186/s12942-018-0165-5)
Supplement: Supplementary file 2 — Additional file 2. List of excluded full text studies with reason. [file 12942_2018_165_MOESM2_ESM.docx]

**Additional file 2: List of excluded full text studies with reason**

|  | **Excluded studies** | **Reason** |
| --- | --- | --- |
|  | Inoue, Y., et al. Neighborhood Characteristics and Cardiovascular Risk among Older People in Japan: Findings from the JAGES Project. PLoS ONE [Electronic Resource] 11, e0164525 (2016). | ‘Accident prone perception ‘is the ASED proxy measurement. |
|  | Sundquist, K., Eriksson, U., Mezuk, B. & Ohlsson, H. Neighborhood walkability, deprivation and incidence of type 2 diabetes: a population-based study on 512,061 Swedish adults. Health Place 31, 24-30 (2015). | ‘Neighbourhood deprivation’ is used as a control in analyses. |
|  | Congdon, P. Estimating diabetes prevalence by small area in England. J Public Health (Oxf) 28, 71-81 (2006). | Methodology oriented paper. |
|  | Mezuk, B. *et al.* Depression, neighborhood deprivation and risk of type 2 diabetes. *Health Place* **23,** 63–69 (2013). | Depression patients are the study population. |
|  | Stoddard, P. J. *et al.* Neighborhood deprivation and change in BMI among adults with type 2 diabetes: the Diabetes Study of Northern California (DISTANCE). *Diabetes Care* **36,** 1200–1208 (2013). | Diabetic patients are the study population. |
|  | Chaikiat, A., Li, X., Bennet, L. & Sundquist, K. Neighborhood deprivation and inequities in coronary heart disease among patients with diabetes mellitus: a multilevel study of 334,000 patients. Health Place 18, 877–882 (2012). | Diabetic patients are the study population. |
|  | Yu, Z., et al. Obesity related metabolic abnormalities: distribution and geographic differences among middle-aged and older Chinese populations. Prev Med 48, 272-278 (2009) | Non-continuous geographic units (i.e prevalence in two cities in the north and south of country are compared, and its urban/ rural cross differences were focussed) |
|  | Chichlowska, K.L., et al. Individual and neighborhood socioeconomic status characteristics and prevalence of metabolic syndrome: the Atherosclerosis Risk in Communities (ARIC) Study. Psychosom Med 70, 986-992 (2008). | No results on discrete CMRFs or its association with ASED. |
|  | Ardern, C.I. & Katzmarzyk, P.T. Geographic and demographic variation in the prevalence of the metabolic syndrome in Canada. Can 31, 34-46 (2007). | No results on discrete CMRFs or its association with ASED. |
|  | Traissac, P., et al. Abdominal vs. overall obesity among women in a nutrition transition context: geographic and socio-economic patterns of abdominal-only obesity in Tunisia. Population health metrics 13, 1-1 (2015). | Obesity results are not presented due to small (1.4%) overall prevalence (waist circumference defined as abdominal adiposity is focussed). |
|  | Jones, M. & Huh, J. Toward a multidimensional understanding of residential eighbourhood: a latent profile analysis of Los Angeles neighborhoods and longitudinal adult excess weight. Health Place 27, 134-141 (2014) | Geographic area based results were not available. Also minimal data on ASED – but three types of neighbourhoods based on ‘social context variables’ were identified in the study. |
|  | Kandala N-B, Manda SOM, Tigbe W, Mwambi H, Stranges S. Geographic distribution of cardiovascular comorbidities in South Africa: a national cross-sectional analysis. Journal of Applied Statistics 2014;41(6):1203-1216 | Age group of the study sample (aged 15 and over) are under the review defined adult age group (18 years and above). |
